# Combining deep learning and fluorescence imaging to automatically identify fecal contamination on meat carcasses

**DOI:** 10.1038/s41598-022-06379-1

**Published:** 2022-02-14

**Authors:** Hamed Taheri Gorji, Seyed Mojtaba Shahabi, Akshay Sharma, Lucas Q. Tande, Kaylee Husarik, Jianwei Qin, Diane E. Chan, Insuck Baek, Moon S. Kim, Nicholas MacKinnon, Jeffrey Morrow, Stanislav Sokolov, Alireza Akhbardeh, Fartash Vasefi, Kouhyar Tavakolian

**Affiliations:** 1 Biomedical Engineering Program, University of North Dakota, Grand Forks, ND 58202 USA; 2 School of Electrical Engineering & Computer Science, College of Engineering and Mine, University of North Dakota, Grand Forks, ND 58202 USA; 3 Electrical Engineering Program, Department of Engineering, SUNY Polytechnic Institute, Utica, NY 13502 USA; 4 Department of Biomedical Engineering, College of Science and Engineering, University of Minnesota, Minneapolis, MN 55455 USA; 5 USDA/ARS Environmental Microbial and Food Safety Laboratory, Beltsville Agricultural Research Center, Beltsville, MD 20705 USA; 6 SafetySpect Inc., 4200 James Ray Dr., Grand Forks, ND 58202-8372 USA

**Keywords:** Engineering, Optics and photonics

## Abstract

Food safety and foodborne diseases are significant global public health concerns. Meat and poultry carcasses can be contaminated by pathogens like *E. coli* and salmonella, by contact with animal fecal matter and ingesta during slaughter and processing. Since fecal matter and ingesta can host these pathogens, detection, and excision of contaminated regions on meat surfaces is crucial. Fluorescence imaging has proven its potential for the detection of fecal residue but requires expertise to interpret. In order to be used by meat cutters without special training, automated detection is needed. This study used fluorescence imaging and deep learning algorithms to automatically detect and segment areas of fecal matter in carcass images using EfficientNet-B0 to determine which meat surface images showed fecal contamination and then U-Net to precisely segment the areas of contamination. The EfficientNet-B0 model achieved a 97.32% accuracy (precision 97.66%, recall 97.06%, specificity 97.59%, F-score 97.35%) for discriminating clean and contaminated areas on carcasses. U-Net segmented areas with fecal residue with an intersection over union (IoU) score of 89.34% (precision 92.95%, recall 95.84%, specificity 99.79%, F-score 94.37%, and AUC 99.54%). These results demonstrate that the combination of deep learning and fluorescence imaging techniques can improve food safety assurance by allowing the industry to use CSI-D fluorescence imaging to train employees in trimming carcasses as part of their Hazard Analysis Critical Control Point zero-tolerance plan.

## Introduction

Unsafe food poses a threat to people worldwide, causing many illnesses and deaths every year^1^. Foodborne diseases are a public health challenge and major contributors to morbidity and mortality. About 600 million cases of foodborne illness and 420 thousand deaths globally are caused by unsafe food each year^2^. According to the US Centers for Disease Control and Prevention (CDC), foodborne illnesses affect millions in the US, causing thousands of deaths and billions of dollars in economic losses annually^3^. Each year about 48 million Americans become sick (1 in 6), 128,000 are hospitalized, and 3000 die from foodborne illnesses^4–6^. The economic burden of foodborne illness was over $15.5 billion in 2013^7^, and the US Department of Agriculture (USDA) stated that this amount was increased by about 13% to $17.6 billion in 2018^8^.

In the United States, the foods that are most likely to become contaminated and therefore unsafe, are raw foods of animal origin^9^. Currently, the meat and poultry industry is the largest segment of agriculture. For instance, in 2017, 52 billion pounds of meat and 48 billion pounds of poultry were produced in the US^10^. With high consumption of meat and poultry comes a high number of human foodborne illnesses. There are numerous foodborne pathogens associated with meat, including *Salmonella* spp., *E. coli*, Campylobacter jejuni, Yersinia, and Listeria monocytogenes. These are the most often detected pathogens that can cause significant public health problems^5,11^. Becoming infected with any of these pathogens can lead to severe diarrhea, vomiting, abdominal cramps, and even death in some cases^5^. Outbreaks of foodborne illness resulting from contamination by these pathogens tend to occur by exposure of the animal carcass to feces, ingesta, and soiled hides during meat processing. According to USDA policy, the regulatory standard for US Food Safety and Inspection Service (FSIS) is “zero tolerance”^12^. “Zero tolerance” is a visual standard by which all surfaces of processed meat and poultry carcasses are required to be free of visible fecal contamination before they can enter the carcass chiller.

Current methods for identifying fecal contamination on carcasses are limited to simple visual examination during meat processing. It is difficult for human inspectors to thoroughly inspect carcasses since some contamination is invisible or barely visible and can be missed. Having a “zero tolerance” requirement for visible fecal contamination on the carcasses creates a problem. The visual inspection method becomes insufficient because of human errors that happen during processing, especially “painting”. In painting, the slaughter knife is not disinfected and cleaned properly of feces or ingesta and is used later to skin the carcass. This error dilutes the fecal contamination, making it difficult to visually detect on the carcass with the naked eye. Another source of error is when fecal contamination is not detected on the lower or upper parts of the carcass, which are more difficult for the inspector to reach and see. To combat meat and poultry contamination, a solution is needed to more effectively inspect meat surfaces during meat processing to overcome potential human error, increase food safety, and increase public confidence in the meat processing system while allowing a fast enough production speed. Although there is no way to ensure that our food is completely secure, new developments in optical fluorescence imaging can provide improved confidence in food processing operations. Fluorescence imaging can play a crucial role in food safety as a swift, precise, and non-destructive technique that is able to detect chlorophyll and its metabolites as well as other fluorophores within fecal matter and ingesta.

There are two problems to overcome when deploying fluorescence imaging technology in meat processing facilities. One of these is the problem of doing fluorescence imaging in a bright ambient light environment. Another is the need for interpretation and analysis of fluorescence images by untrained operators. New technologies in Light-emitting diode (LED) illumination and image sensors have been developed to overcome the bright ambient light problem, and modern machine learning algorithms can be applied to automate image analysis and provide immediate feedback to inspectors and meat cutters.

In this study, we employ a fluorescence imaging device developed by SafetySpect and two state-of-the-art deep learning algorithms, including EfficientNet and U-Net, to first discriminate the video frames with fecal residue on meat surfaces and then accurately segment and identify the corresponding areas of fecal contamination in the image. The handheld automated imaging inspection device (CSI-D), created by SafetySpect^13^, offers mobility and flexibility for fluorescence-based contaminant detection on both food products (e.g., carcasses) and equipment surfaces in meat processing facilities under bright ambient light. Visualization by fluorescence emission has excellent potential for food safety procedures. However, fluorescence imaging, like many other imaging techniques, delivers video or images that need to be interpreted to be used for live visual inspection. These images need automated methods to identify contamination and make decision-making easier for human inspectors and meat cutters excising the contaminated areas from meat. This is the next level of safety that needs to be developed and which we address in this research.

Research in applications of image processing and machine learning algorithms over the last few years has rapidly increased in the area of food safety and food security, especially in the meat processing industry^14,15^. Researchers evaluated the quality of 16 types of pork and poultry using image processing techniques, including segmentation and histogram-based analysis^16^. Another study used 4-bit monochromatic images of meat cuts to extract features using image processing techniques from fatty regions of meat images and then used neural network and multiple regression analysis to grade the meat quality^17^. To assure meat safety, several studies used threshold-based algorithms on fluorescence images to detect fecal contamination on beef and poultry meat surfaces^18–20^. However, most of these techniques are prone to error and are not reliable enough to be applicable in the real world for the following reasons.

Many of these experiments were conducted in a laboratory environment with low ambient light intensity and on surfaces that were dark as opposed to the stainless steel and bright plastic surfaces typically found in meat processing plants. The meats being measured were mostly fixed in position as opposed to the constant motion of carcasses in meat processing plants. Additionally, many of the techniques discussed required predefined feature segmentation based on feature shape or color. These identified features are then used to extract the meaningful information for further analysis for classification. Several studies used thresholding-based algorithms to detect the contamination regions in which determining the optimum value of the threshold level is very challenging and can cause both false positive and false negative outcomes due to varying ambient light intensity and the variety of background surfaces found in meat processing plants. This is why more sophisticated and reliable algorithms are needed to fill the gap in current analysis methods.

During the past few years, deep neural network (DNN) algorithms, and more specifically, convolutional neural network (CNN) algorithms, have grabbed much attention in many fields as state-of-the-art machine learning methods^21,22^, and certainly in food safety-related technologies^23,24^. One of the main advantages of CNN models is that they can automatically extract important and meaningful information from images and videos and learn from them without image feature extraction by human-derived techniques. The learned weights of a trained CNN model can also be saved and reused with a new dataset without re-training the model^25^. DNN algorithms have a wide variety of applications in the food domain, including food recognition and classification, food calorie estimation, food supply chain monitoring, food quality detection, food contamination detection, etc.^23,26^. Although applications of DNN algorithms have been studied in several food-related domains, to our knowledge, there is no study where the main focus is on the detection and segmentation of fecal contamination on meat surfaces.

## Material and methods

### Fecal contamination measurement technology

In this study, to collect the fecal contamination information, we used a handheld contamination, sanitization inspection, and disinfection device (CSI-D). One of its capabilities is the detection of organic residues in meat processing facilities. The CSI-D device integrates illumination, imaging, data processing, and display into a single portable device. Its illumination module is composed of 270 nm and 405 nm light-emitting diode (LED) arrays, a suitable heatsink for dissipation of heat from the LEDs and driver circuits. In addition, the device incorporates a Wi-Fi transmitter for streaming captured video to supplementary monitoring devices like tablets and cell phones.

**Figure 1:**
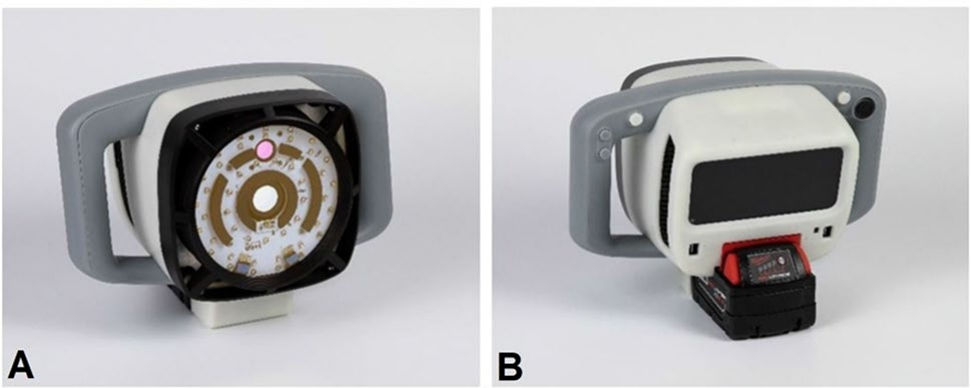
CSI-D device. (**A**) Front view. (**B**) Rear view.

During imaging mode, the LED arrays are automatically pulsed—switched off and on in rapid sequence through electrical signals to enable background image subtraction. For fluorescence imaging, there are two cameras: an RGB camera and a UV camera, that are controlled by a processing unit that initiates capture of fluorescence images. The RGB camera captures images of organic residues, and the UV camera captures images of saliva and respiratory droplets and certain other organic residues. CSI-D is also equipped with lenses and spectral bandpass filters that pass specific wavelengths emitted by contamination fluorescence. Figure 1 shows the CSI-D device.

### Data collection

We collected data at three meat processing facilities (two cattle, one sheep) in North Dakota, USA. Before starting data acquisition, there was a detailed discussion with the owners and local meat inspectors to better understand the current methods used in their facilities, including any perceived needs or shortcomings. SafetySpect's CSI-D device was used to record videos from processed raw meat carcasses, initially under the direction of a retired federal meat inspector and later without direction. If we found anything suspicious during the scan, we asked the inspector to review it. We scanned fourteen beef and six sheep carcasses on the left and right sides of the carcasses after skinning and before they were placed in the chiller.

In addition to the video recording of the scans, we also created a video record of the procedure with the meat inspector using a GoPro camera. We also secured samples of meat, fat, and sheep feces for further measurements in the lab at the University of North Dakota.

All measurement scans were captured with a resolution of 1024 × 768 and at 24 frames per second (FPS). In total, 1 h and 7.5 min of video scans were used for further analysis.

**Figure 2:**
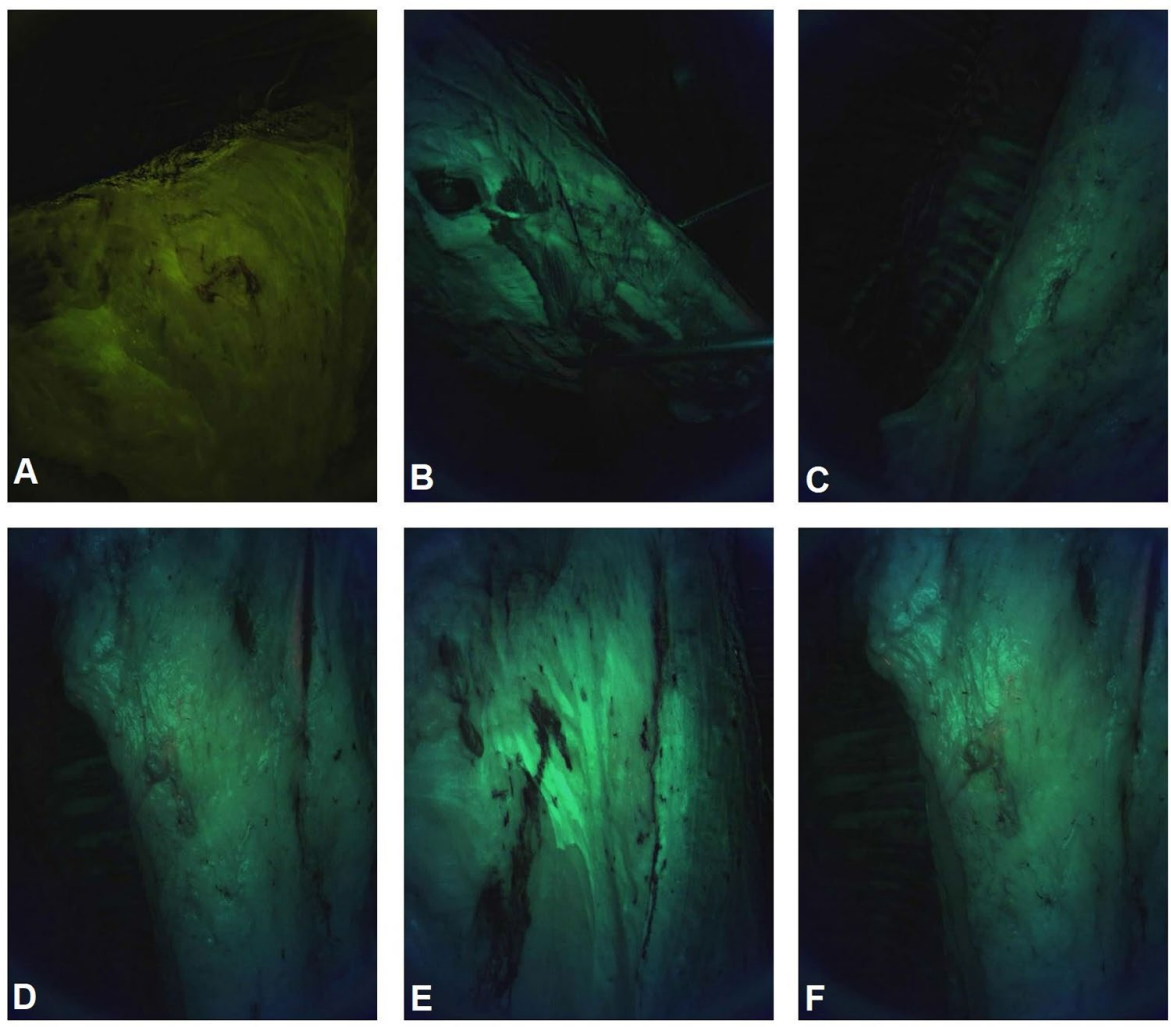
Six CSI-D fluorescence images of clean meat surfaces (**A**–**F**).

**Figure 3:**
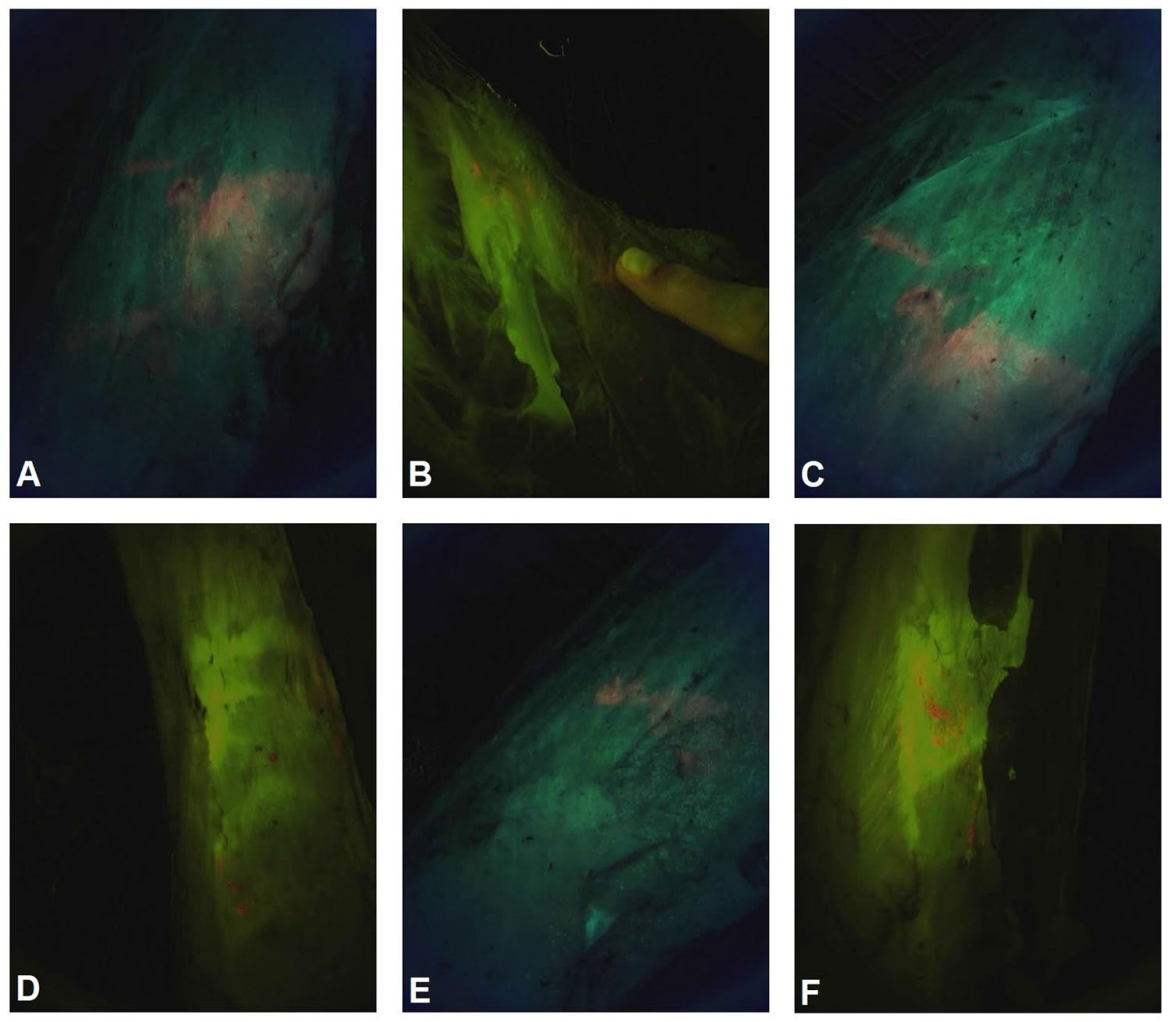
Six CSI-D fluorescence images of meat surfaces with fecal contamination (**A**–**F**).

Figure 2 shows examples of image data collected from clean meat surfaces, and Fig. 3 shows examples of image data from meat surfaces contaminated by fecal matter.

### Methodology

For this study, we had two aims. The first aim was to differentiate the frames showing fecal contamination on meat surfaces (*contamination*) versus the frames with no contamination on meat surfaces (*clean*) using a DNN model. The second aim was to identify the specific areas of fecal contamination using a semantic segmentation algorithm on the frames already classified as *contamination*.

#### Deep learning model architecture

In this study, a CNN model is used to classify the captured video frames from the CSI-D device into two classes of *clean* and *contamination*. To do so, first, all the captured videos were converted to the frames, and then each frame that showed contamination was labeled as *contamination*, and each frame without any contamination was labeled as *clean*.

We then employed a state-of-the-art CNN model (EfficientNet) for classification between *clean* and *contamination* frames.

The EfficientNet model was proposed by Google researchers^27^, and their main idea was that balanced scaling up of a CNN in terms of depth, width, and input image resolution could lead to better accuracy and efficiency. Their empirical investigation revealed that balancing such dimensions resulted in a more accurate outcome. They used a neural architecture search^28^ and designed a baseline network (EfficientNet-B0), and proposed a compound scaling method in which all network dimensions (width, depth, and image resolution) can be uniformly scaled using a set of predefined scaling factors to generate a family of EfficientNets (B1–B7).

**Figure 4:**
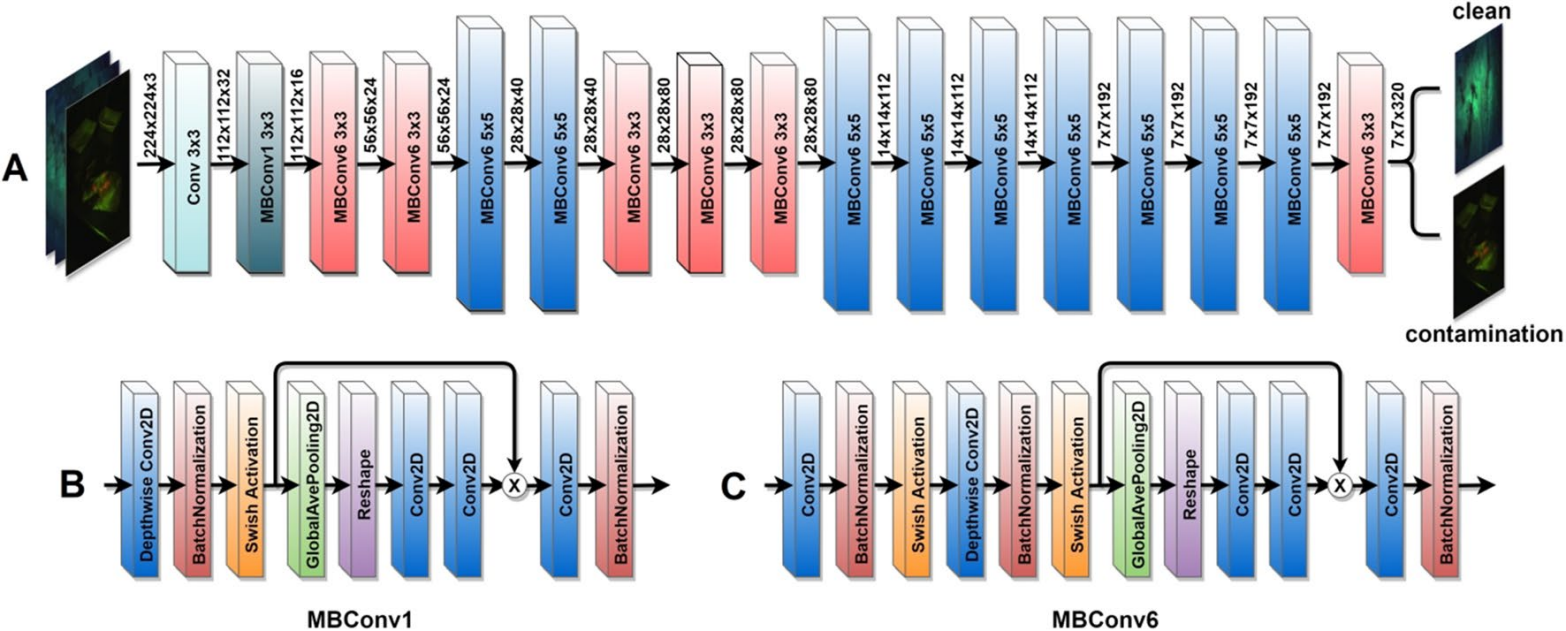
(**A**) A concise representation of the EfficientNet-B0 model. (**B**) The building blocks of MBConv1. (**C**) The building blocks of MBConv6.

We used EfficientNet-B0, and Fig. 4 depicts a condensed schematic representation of this model.

As can be seen, the main building block of EfficientNet-B0 is a mobile inverted bottleneck convolution (MBConv)^28,29^, which is slightly modified by adding squeeze-and-excitation optimization blocks^30^. Each MBConv block relies on depthwise convolutions^31^ and shortcut connections between the blocks. The squeeze-and-excitation enhances the network's representative power by explicitly modeling interdependencies among channels, resulting in dynamic channel-wise feature recalibration. Unlike typical two-dimensional (2D) convolutions, depthwise convolutions can apply a single convolutional filter to each input channel and not only decrease the number of parameters and computational cost but improve the network's representational efficiency.

The input image size for EfficientNet-B0 should be (224, 224, 3), and the model expects the inputs to be floating pixel tensors with values in the range of 0–255. The input images pass through a 2D convolutional layer with 32 filters and a kernel size of 3 × 3. Then the images flow through sixteen other MBConvs, including one MBConv1 and fifteen MBConv6 with different filter and kernel sizes. It is worth mentioning that each convolutional layer is followed by a batch normalization^32^ along with a swish activation function^33^. The batch normalization by smoothing the optimization landscape can make the gradients more stable and predictable, making optimization faster and more effective, and can speed up the training process^34^.

The last block of the EfficientNet-B0 is composed of another convolution layer followed by another batch normalization and the swish activation function. More specifically, a 1 × 1 convolution layer^35^ is used in the final block, which enables the network for channel-wise pooling or cross-channel downsampling. By doing so, the 1 × 1 convolution layer functions as a projection layer that aggregates information across channels and reduces the dimensionality by decreasing the number of filters while introducing non-linearity and keeping essential, feature-related information.

Given that the first aim of our experiment was binary classification between *clean* and *contamination* frames, we employed a sigmoid activation function as the output activation layer. The sigmoid function is a nonlinear activation function that transforms the values between 0 and 1, and in our case, its output represents the probabilities that an input frame belongs to the *clean* and *contamination* classes.

Next, the EfficientNet-B0 model needs to be trained to recognize and classify the input frames correctly, and the model error for the prediction of the correct classes needs to be minimized. A loss function is needed to compute the error and quantify the performance of the model. Since our task is a binary classification, we used Binary Cross-entropy as our loss function. Binary Cross-entropy calculates the cross-entropy loss between the predicted labels and true labels, which can be 0 or 1. By diverging the predicted labels from true labels, the cross-entropy loss will be increased. The Binary Cross-entropy Eq. (1) is as follows:

1 $$Loss= -\frac{1}{N}\sum_{i=1}^{N}{y}_{i} \times \text{log}\left(p\left({y}_{i}\right)+\left(1-{y}_{i}\right)\times \text{log}\left(1-p\left({y}_{i}\right)\right)\right)$$

where N is the number of training samples, ${y}_{i}$ denotes the true label, $p\left({y}_{i}\right)$ represents the predicted probability of class 1 (in our case, *contamination*) and $1-p\left({y}_{i}\right)$ is the predicted probability of class 0 (*clean*).

To minimize the loss function and improve the model performance, an optimization algorithm is needed. In this study, we used the Adam optimizer, a gradient-based optimization of stochastic objective functions, which adaptively estimates the first and second-order moments^36^. In other words, the Adam optimizer individually adjusts the learning rate of the parameters using the estimation of the first and second moments of the gradients.

#### Contamination segmentation

The second aim of this research was to segment the regions with fecal contamination after finding and classifying the video frames as *contamination* using the model described above. The accurate segmentation of areas contaminated by fecal matter can be very beneficial for the following reasons. The first and foremost reason is that finding *contamination* frames does not always lead to finding a contaminated area on the meat surface. In many cases, the fecal matter on the meat surface is very small, and finding areas of contamination even after detecting the corresponding frame could be challenging during a live inspection process.

Since carcass inspection must comply with the "zero tolerance" standard, segmenting and pseudo-coloring these areas can make it more straightforward for inspectors to recognize them and not miss any contamination. Another reason for pseudo-coloring is that the segmentation can improve inspector discrimination during the monitoring process. During the inspection, there might be several contaminated regions of different sizes or colors at the same time on meat surfaces, and without accurate segmentation, the inspector might miss some contaminated areas. Developing a model for accurate segmentation of the fecal contaminated areas on meat surfaces may be crucial. Further, the segmentation of the fecal contaminated area can help the training process of new FSIS inspectors in recognition of the possible fecal contamination areas and in trimming carcasses as part of their Hazard Analysis Critical Control Point (HACCP) zero-tolerance plan.

In the past, fecal contamination segmentation on the surface of different types of meats and vegetables has been tackled mainly using threshold-based algorithms. However, such algorithms are prone to error because they are sensitive to ambient light intensity, and determining an appropriate thresholding level can be challenging. In real-world situations, the presence of other objects with similar pixel intensity in the background can also lead to false positives^18,20,37,38^.

In this study, we used the semantic segmentation method to precisely segment fecal contaminated regions on meat surfaces. In semantic segmentation, a pixel-level annotation assigns a class to each pixel of an image. Then a deep CNN is first trained to learn to classify pixels according to their corresponding classes (in our case, *clean* and *contamination*), and then the trained model is used to predict the class of each pixel for the unseen data (test set). To build the semantic segmentation training and testing datasets, we employed MATLAB Video Labeler for pixel-wise labeling of each video frame. The Video Labeler provides a straightforward way to label ground truth data in a video or image sequence by marking rectangular or polyline region of interest (ROI) labels, pixel ROI labels, and scene labels.

Since the aim was to segment the fecal contamination regions on meat surfaces, the ground truth labels consisted of two classes of *contamination* and *background*, and all pixels were labeled accordingly. We labeled every pixel of 55,114 frames that had already been classified as *contamination* in the previous DNN classification described above. It is worth mentioning that the ground truth labeling process was conducted by two labelers and reviewed by two supervisors who were present during the data collection.

**Figure 5:**
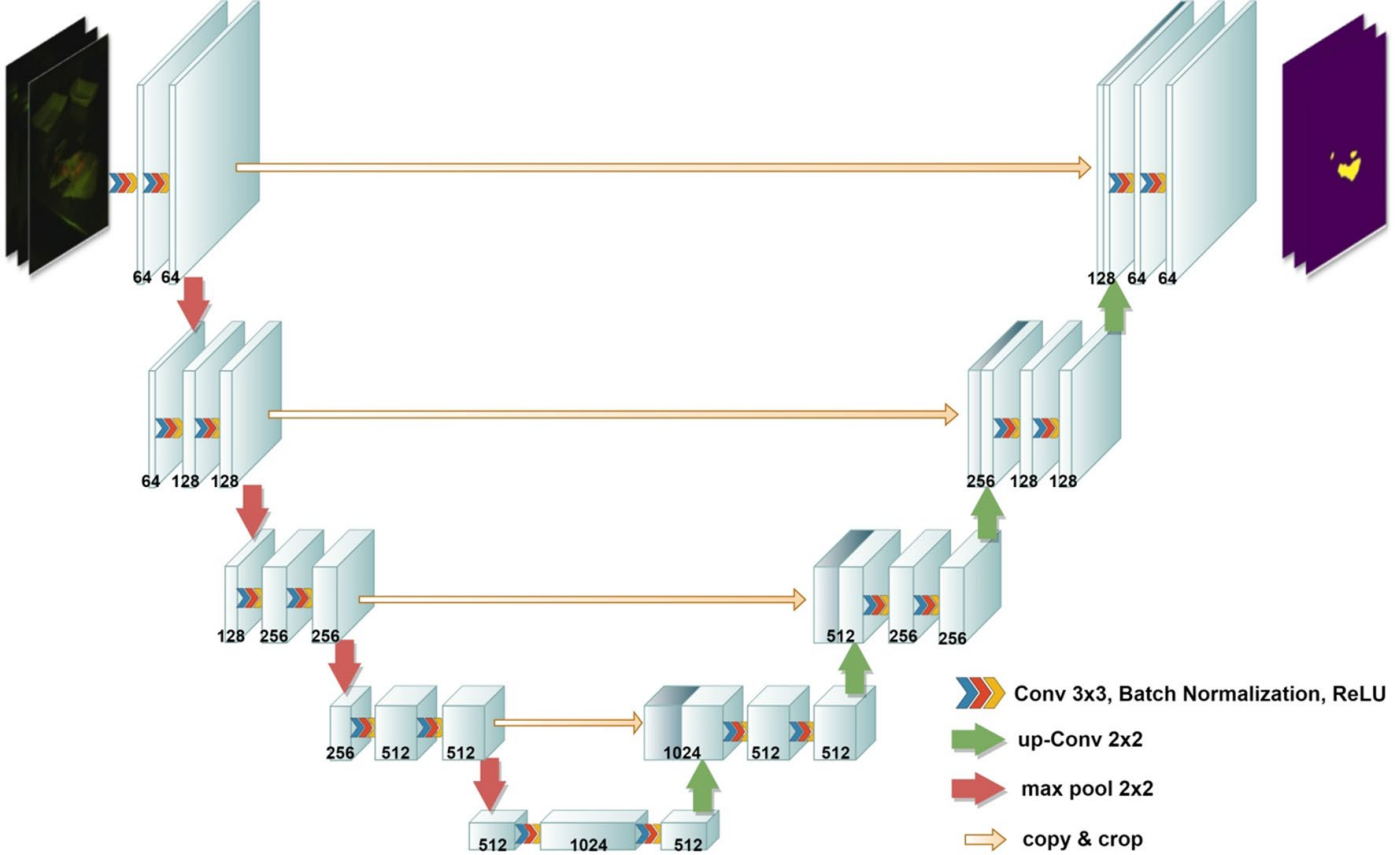
U-Net architecture.

To accomplish the semantic segmentation task, we used U-Net, a CNN model initially developed for biomedical image segmentation^39^. In the U-Net architecture (Fig. 5), the input images pass through three sections, including a contraction path (left side), bottleneck, and expansion path (right side). Before feeding the images to the contraction path, we first resized the video frame to 512 × 512 × 3 pixels and then rescaled the range of each pixel intensity from [0, 255] to [0, 1] to speed up the model convergence learning process.

The contraction path also called the downsampling path, comprises two 3 × 3 convolutions that are repeated several times. These two convolution layers are followed by a rectified linear unit (ReLU) and a downsampling layer composed of a 2 × 2 max-pooling process with a stride of 2. The number of feature channels (filters) at each downsampling step was doubled, allowing the architecture to learn more complex structures.

The data then passes through the bottleneck, a nonlinear dimensionality reduction layer, which is the nethermost section of the U-Net architecture and operates as a bridge between the contraction and expansion paths. The data then flows through the expansion path, each step of which is similar to the contraction path. It comprises repeated application of two 3 × 3 convolutions, followed by a 2 × 2 up convolution layer that reduces the number of feature channels by half to maintain the architecture's symmetry. It is worth mentioning that the feature maps of each of the two-convolution layers in the contraction path are concatenated with the feature maps of the same layer in the expansion path. By doing so, the high-resolution feature maps from the contraction path are concatenated with the upsampled features of the expansion path, which can improve the model's ability to learn and localize the representations. It is also worth noting that after each convolution layer, we used a batch normalization to solve the problem of internal covariate shift^32^.

Finally, since our task is a binary classification at the pixel level (*contamination* vs. *background*), a sigmoid function is used for the output layer. Also, binary cross-entropy was employed as the model loss function, and the Adam optimizer was employed to update model weights and learning rate for minimizing the loss.

### Experimental setup

When evaluating the models for classification between *clean* and *contamination* frames, and also when evaluating the image segmentation of the fecal matter on meat surfaces, all codes were implemented using the Keras framework (open-source library for solving machine learning problems). We used the Tensorflow-GPU v2.6.0 backend on a GPU-enabled workstation with an NVIDIA GeForce GTX 1080 FTW 8 GB GDDR5. The experiments were conducted with Windows10 as the operating system.

## Results and discussion

### Model performance for clean vs. contamination classification

The classification between *clean* and *contamination* was carried out on a total of 108,296 frames, including 53,182 *clean* frames and 55,114 *contamination* frames. We chose 70% of frames at random as our training set, 20% for validation, and 10% of the frames as the test set to evaluate the performance of our classification model. Since deep learning models result in higher performance in the presence of rich and sufficient data, we used some data augmentation methods such as random flip and rotation to improve our model's accuracy and generalization.

We trained and validated our model over 150 epochs by choosing binary cross-entropy as the loss function and Adam optimizer with a learning rate of 0.001. We also set the batch size to 32. Batch size is the number of propagated frames through the network in one iteration during the model training.

Our model performance was evaluated using the six metrics of accuracy, precision, recall, specificity, F-score, and area under the curve (AUC). The first five metrics are defined in Eqs. (2) and (3):

2 $$\text{Accuracy}= \frac{(\text{TP}+\text{TN})}{(\text{TP}+\text{TN}+\text{FP}+\text{FN})},\text{ Precision}= \frac{\text{TP}}{\text{TP}+\text{FP}}, \text{Recall}= \frac{\text{TP}}{\text{TP}+\text{FN}}$$

3 $$\text{Specificity}= \frac{\text{TN}}{\text{TN}+\text{FP}}, {\text{F}}_{\text{score}}=2\times \frac{\text{Precision}\times \text{Recall}}{\text{Precision}+\text{Recall}}$$

where TP, TN, FP, and FN denote true positives, true negatives, false positives, and false negatives, respectively. In addition, we used the area under the curve (AUC) of the receiver operating characteristic (ROC), as an indicator of the model's ability to separate the two classes.

**Figure 6:**
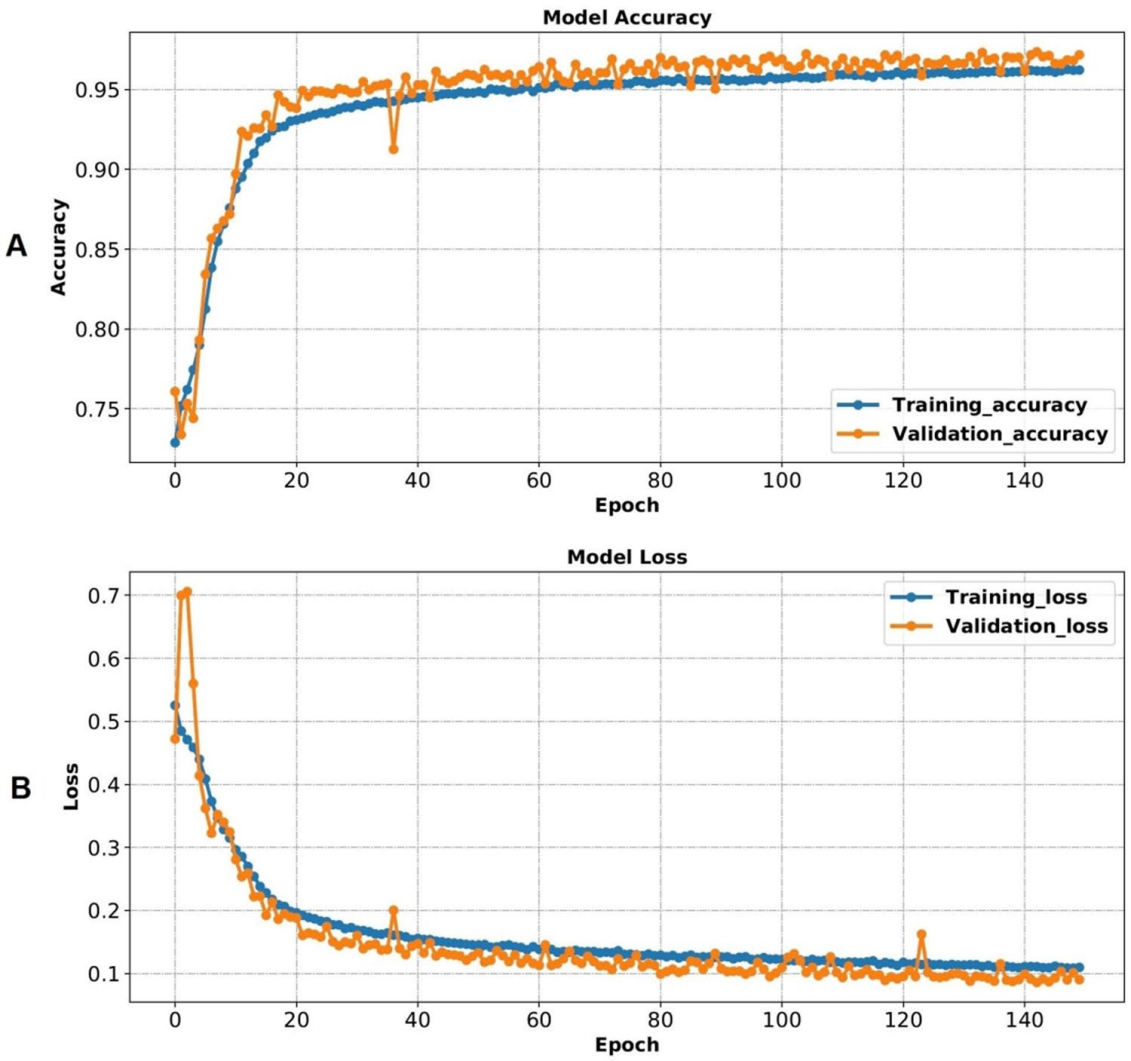
(**A**) The model accuracy during training and validation. (**B**) The model loss during training and validation.

**Figure 7:**
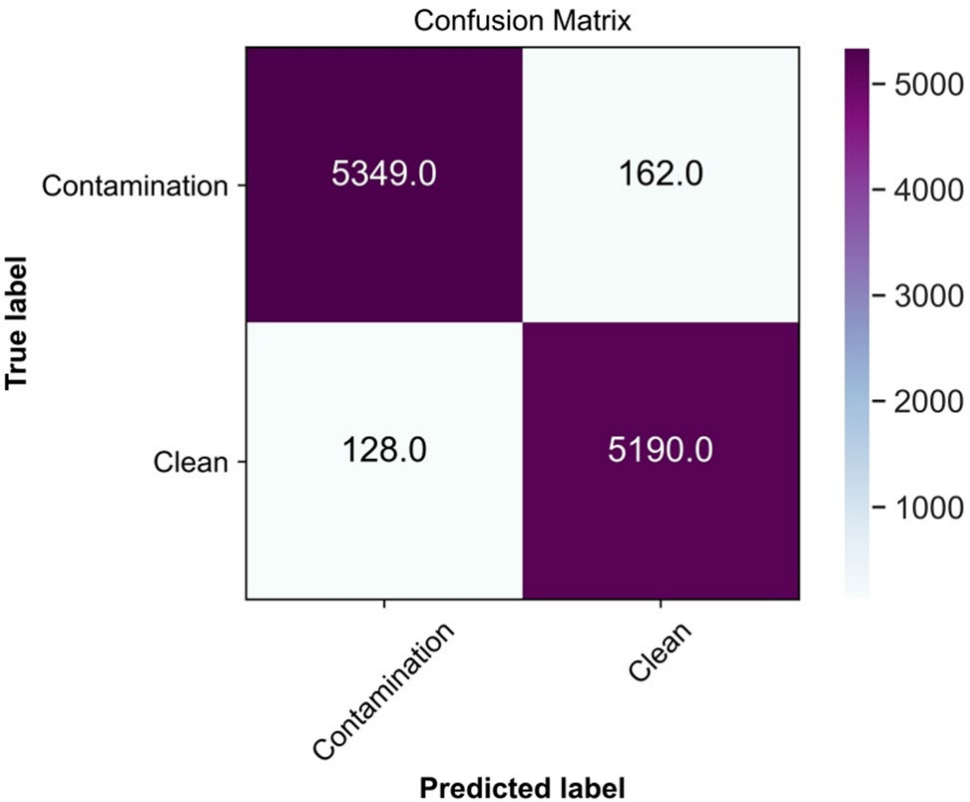
The confusion matrix of the model when applied to the test set.

As shown in Table 1, the model could successfully discriminate between the two classes of *clean* and *contamination* with an accuracy of 97.32%, precision of 97.66%, recall of 97.06%, specificity of 97.59%, F-score of 97.35%, and AUC of 99.54%. Figure 6 shows the accuracy and loss of the model during training and validation. Figure 7 illustrates the confusion matrix of the model on the test set, which is used as a visual evaluation tool in classification. The rows of the confusion matrix show the actual class for *clean* and *contamination*, and the columns represent the predicted label of each class.

**Table 1:** Performance of the EfficientNet-B0 for discrimination between *clean* and *contamination* frames.

Evaluation metrics	Accuracy (%)	Precision (%)	Recall (%)	Specificity (%)	F-score (%)	AUC (%)
	97.32	97.66	97.06	97.59	97.35	99.54

### Model performance on fecal contamination image segmentation

The segmentation of areas of fecal matter in meat surface images is used to determine the presence and the exact location of contamination. The model was trained, evaluated, and tested on the 55,114 frames already labeled as *contamination* in the classification section. Like the previous section, 70% of the data was chosen for training, 20% for validation, and 10% as the test set.

Our semantic segmentation model was trained and validated over 100 epochs. Like our classifier, we chose binary cross-entropy as the loss function, the Adam optimizer with a learning rate of 0.001, and a batch size of 32.

The model performance was evaluated using the same metrics as described above. However, instead of accuracy, we used another metric, intersection over union (IoU), to assess the model segmentation performance. The rationale for not using accuracy is that our semantic segmentation model is a pixel-wise binary classifier (*contamination* vs. *background*). Since the fecal matter usually covered just a tiny portion of the frames, the accuracy would be consistently over 99%, making it not useful for evaluating the model performance.

IoU, also known as Jaccard Index, is a well-known metric for assessing how accurately a segmentation method can segment the ROI compared to ground truth segmentation. The IoU metric quantifies the percentage of overlap between the ground truth and the model prediction.

Equation (4) shows the IoU definition.

4 $$\text{IoU}= \frac{\text{TP}}{(\text{TP}+\text{FP}+\text{FN})}$$

Our semantic segmentation model segmented the fecal matter on meat surfaces with an IoU of 89.34%, precision of 92.95%, recall of 95.84%, specificity of 99.79%, F-score of 94.37%, and AUC of 99.89% (Table 2).

**Table 2:** Performance of the U-Net for segmentation of fecal matter in meat surface images.

Evaluation metrics	IoU (%)	Precision (%)	Recall (%)	Specificity (%)	F-score (%)	AUC (%)
	89.34	92.95	95.84	99.79	94.37	99.89

**Figure 8:**
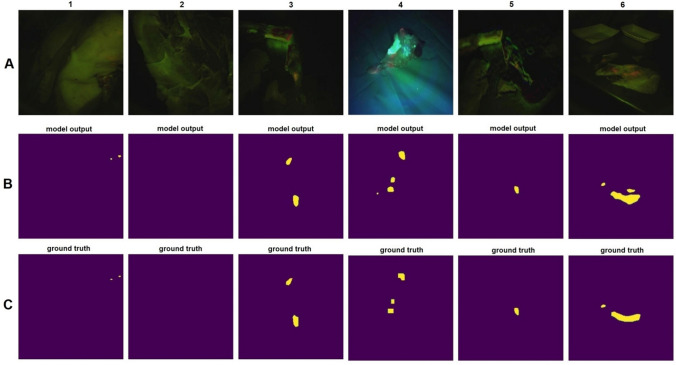
Performance of the semantic segmentation method on some randomly selected test frames. (**A**) The input frames to the semantic segmentation model. (**B**) Segmented image output by the model. (**C**) The ground truth segmented image by human experts.

For better visualization to show how accurately our model can segment fecal matter areas in meat surface images, we show six sample images that were randomly chosen from the test set (Fig. 8). The first row of Fig. 8 shows the input frames to be analyzed using the semantic segmentation model. The second row is the segmented image output by the model, and the third row shows the ground truth segmented image where a human expert has labeled each pixel.

Comparing the model output with ground truth shows that our model can segment fecal matter on meat surfaces with high accuracy. Interestingly, in columns four and six, there are two small areas that the model segmented as fecal matter but were not labeled in the ground truth by our human experts. A closer look at the input frames reveals that those areas were chosen correctly and precisely as fecal contamination by the model, and the three human experts, when reviewing the images, agreed that they had missed these tiny spots during labeling of the images.

The goal of this research is to develop a system and software that can be used to make processed meat safer for consumers. Detecting contamination can be extremely challenging in production environments that have a lot going on at once. We need to overcome fluorescence imaging issues caused by bright ambient light, continually changing shadows as carcasses move about the processing area, and often high-speed production requirements. It is difficult for a human to recognize these kinds of contamination without some form of automated analysis. In this paper, we have demonstrated the ability of state-of-the-art machine learning models to quickly analyze the presence of contamination with a portable handheld carcass scanning system. This system has been well received by the meat processing personnel working on the floors of the meat processing plants where we have been testing.

The detection of contaminated regions using CSI-D is beneficial because action can be taken immediately to cut away meat with fecal contamination from the carcass and help the facility meet the "zero tolerance" standard for fecal contamination that is required by the USDA FSIS. CSI-D can also help train new processing plant staff and FSIS inspectors in locating contamination that might be difficult to see during visual inspections. Although fluorescence-based imaging can be beneficial, the detection of fecal matter on meat surfaces can be challenging because the fecal contamination may be very small, there may be multiple tiny spots on a carcass, and the contamination may be a very faint smear, making detection difficult during the live inspection. The contaminated regions that fluoresce can be of different sizes and of varying shades or hues depending on the color of the meat or fat in the background and the mix of fluorophores being detected as well as their concentration. This can increase the risk of missing some contaminated areas by inspectors. That is why developing a model for accurate detection of the contaminated areas on meat could be crucial. The inspection process will be more straightforward for inspectors to recognize fecal residue on meat surfaces if we have an accurate and reliable model that can highlight contamination.

The model we have developed provides a substantive improvement in the ability to detect even faint indications of fluorescence of fecal contamination.

## Conclusion

These results demonstrate that applying deep learning algorithms in the food inspection domain can provide higher levels of food safety assurance. State-of-the-art deep learning algorithms can be used with new fluorescence imaging systems for detecting fecal residues on meat surfaces to keep our meat and poultry safer than is possible with conventional visual inspection alone.

We have presented a contamination detection technique based on deep learning that automatically identifies unclean meat surfaces contaminated with fecal matter in a fluorescence video image. We have also shown how video image processing and new LED illumination and imaging sensor can overcome the problem of detecting contamination using fluorescence under the bright ambient light found in meat processing facilities. Our methods can also segment and highlight areas of fecal residue in images of contaminated meat surfaces for easy identification and trimming from carcasses in real-time during meat processing.

We employed a state-of-the-art deep convolutional neural network architecture named EfficientNet-B0 on 108,296 images extracted from videos and labeled as "clean" and "contamination", using 70% of the images for training and 20% of the images for validating the model. When tested on the remaining 10% of the images, classification results yielded a 97.32% accuracy (97.66% precision, 97.06% recall, 97.59% specificity, 97.35% F-score, and 99.54% AUC). We segmented areas of fecal residue in images classified as "contamination", using the U-Net semantic segmentation algorithm. Segmentation results from 55,114 "contamination" frames yielded an intersection over union (IoU) score of 89.34% (precision of 92.95%, recall of 95.84%, specificity of 99.79%, F-score of 94.37%, and AUC of 99.89%).
